# The fine line between deliberate play and deliberate practice within talent development in grassroots football: evidence from a one-year longitudinal case study

**DOI:** 10.3389/fspor.2026.1799878

**Published:** 2026-04-27

**Authors:** Dag Vidar Hanstad, Eivind Å. Skille, Bjørn Tore Johansen, Anna-Maria Strittmatter

**Affiliations:** 1 Department of Sport and Social Science, Norwegian School of Sport Sciences, Oslo, Norway; 2 Department of Physical Education and Sport, University of Inland Norway, Elverum, Norway; 3 Department of Sport Science and Physical Education, University of Agder, Kristiansand, Norway; 4 School of Health Sciences, Örebro University, Örebro, Sweden

**Keywords:** deliberate play, deliberate practice, grassroots football, talent development, training diary

## Abstract

**Introduction:**

While former studies into talent development in football have considered elite clubs and retrospective studies, this single-case study provides detailed data on the training activities of a 13-year-old male football player in a grassroots club who later became a professional at the highest level in Norway.

**Methods:**

An intrinsic longitudinal single-case study design was adopted to develop an in-depth, contextually grounded understanding of the load and forms of physical activity within and beyond organized football as performed and experienced by one specific player over the course of a full year in a grassroots football environment. The empirical material includes daily logging and categorization of all activities in a training dairy, with notes provided by the player - and supplemented with in-depth semi-structured interview with the player.

**Results:**

The dataset comprises 733 h of activity over one year, the majority of which consisted of organized football-specific activity (team training: 196 h; matches including warm-up: 84; private football academy training: 110). In addition, the player engaged in 157 h of unorganized football (alone: 79; with friends: 78), sport-related school activities (physical education: 59; unorganized football play in the schoolyard: 28), and 99 h of individual non–football-specific training such as strength training, injury-prevention exercise, and recreational activities including cross-country skiing and family leisure.

**Discussion:**

This case from grassroots football demonstrates substantial variation between deliberate practice and deliberate play. Two categories that are conceptually distinct yet appear equally important in the developmental pathway. At the same time, some training forms represent a blend of play and practice. For instance, unorganized football with friends in this study displays several characteristics typically associated with deliberate practice, whereas team training with a coach also contains elements of play.

## 1 Introduction

Over the past decades, talent development has become an increasingly extensive field of research, spanning multiple directions. The concept of talent remains contested because it is difficult to define and is used in many ways (1, 2). Baker et al. (3) ask whether it is time to remove “talent” from discussions about athlete development altogether. They argue that generic use is both unhelpful and misleading. Nevertheless, they conclude that the concept should be retained in sport but reframed to link individual development to the micro- and macro-environments that shape athletes' learning, training, and growth. In doing so, talent development can create opportunities for a broader range of athletes beyond those identified as “talented” at an early age. This aligns with a more holistic and ecological approach to talent development (4–7).

Identifying and developing the most “talented” athletes is a concern across all sports. In football, one of the world's most popular sports, talent development receives particular attention, as investments can yield enormous returns for clubs, with the buying and selling of players forming a central part of the business model (8). Increasingly, this development takes place through elite clubs' academies, where players are often recruited while they still are children. Sweeney et al. (9) refer to this as premature professionalization. For a small minority of players, this marks the beginning of a long process culminating at the highest professional level. For the vast majority, however, the dream ends abruptly. Other areas of research on football talent development concern the balance between diversified and sport-specific training (10–12), the risks of overtraining and injury (13), relative age effects (14), socioeconomic status (15), birthplace effects (16), the role of coaches (17), early selection (2), and privatization of the activity (18).

The “academy pathway” is not the only route. It is still possible to progress from grassroots football, for example through local clubs or community teams (19). Despite all these elements, one fundamental fact remains: to become proficient in any sport, including football, young athletes must train a lot. The aim of this paper is to examine the load and nature of physical activity both inside and outside organized football to shed light on one player's talent development pathway in grassroots context. In an intrinsic, longitudinal single-case study, we take a closer look at the training data for *one* player in a team of 16 boys. The 13-year-old footballer, who we assign the pseudonym Jason (now 23 years old), became professional at the highest level in Norway, including one game for Norway's junior national team (U19).

The study contributes to knowledge on talent development processes, as it is based on unique empirical insights and is situated within a context that has been mainly neglected in existing football talent development research: grassroots football. By doing so, the study fills three gaps in research literature. First, by providing and analyzing day-by-day data from an entire year, it differs from retrospective studies, with all their uncertainties, which have dominated the field (20–22). Second, the paper offers detailed insight into the wide variation of activities within categories such as “team practice”, “match play” and “free play”, which have often been treated as relatively homogenous units in retrospective research (23–25), and therefore combine time-based summaries with illustrative examples of typical training sessions and training weeks. Third, most of the empirical studies on football talent development processes have been conducted in professional academies or other institutionalized high-performance environments, with relatively little attention to grassroots football settings (26, 27). In contrast, the present study is situated within the grassroot football. The mix of activities and structural conditions differs markedly from those typically described in elite academy research.

The study lies within Norwegian grassroots football. The Norwegian Football Federation (NFF) is one of 55 national sport associations under the umbrella of the Norwegian Olympic and Paralympic Committee and Confederation of Sports (NIF). NFF has 400,000 members, more than twice as many as number two and three (Golf and Handball). Norway has a set of explicit regulations for children up to 12 years of age that organizations at all levels must follow (28). In t*he children's rights in sport,* it is stated that children have the right to experience mastery and to learn many different skills. They should also be given opportunities to experience variation, practice, and teamwork. *The provisions of children's sports* regulate competitions. The competitive element should be toned down, and results lists, tables and rankings *cannot* be used in competitions before children turn 11 years old. Before they turn 12, children cannot participate in Norwegian Championships, European Championships, World Championships or equivalent competitions (21).

Nevertheless, sport for children and youth is under pressure due to increasing specialization, professionalization, and privatization (29). Among other things, virtually all clubs in the top two divisions (men's football) have their own academies. Since 2017, the organization “Norsk Toppfotball” (Norwegian Elite Football) has had its own academy classification, where one of the criteria for success is that clubs develop their own players (30). NFF has its “national team school” (Landslagsskolen) where players are selected from the age of 12. Alongside organized football, many private and commercial academies have emerged, offering high-quality training in small groups for ambitious children (18).

While elite clubs have their own academies, it is still possible to follow the “traditional route”. For example, Erling Haaland, one of the best strikers in the world (today in Manchester City), was part of a team in a club at level two during childhood and adolescence (27, 31, 32). A similar pathway was followed by Martin Ødegaard, the current team captain for Arsenal and Norway's national team. He was not part of an academy during childhood but had a massive load of unorganized football from the age of five years onward (Ødegaard was anonymized in the scientific paper, but the authors used his full name when communicating their findings in the Norwegian media (33).

In Norway, children's sports are typically coached by parents, starting at five or six years. This was also the case for the player under scrutiny, Jason. From his first year in primary school, the team conducted one organized training session per week up to sixth grade, supplemented by one match per week from May to October. Beyond this, there was extensive unorganized football in various forms. He also participated in a variety of organized training sessions across other sports, including handball, cross-country skiing, table tennis, and chess. He was involved in these activities for two or three years – characterized as “sampling”, that is, “trying out” rather than “committing to” different sports (34). In Norway, this is typical across sports (35). During the year under scrutiny for this study (at the age of 13) he did not participate in any other (organized) sports than football.

## 2 Conceptual framework

### 2.1 Deliberate practice and deliberate play

There are two dominant perspectives applied to analyze training in youth football: deliberate practice (36), and deliberate play (37). In this paper, we use these concepts as analytical lenses to characterize different ways of training within Jason's everyday activity pattern (38–40).

In line with deliberate practice, training is highly specified and designed and supervised by a coach. The coach uses his or her knowledge to help the player identify specific weaknesses and design tasks slightly beyond their current capabilities. The primary goal of deliberate practice is not winning a particular game or competition, but targeted performance improvement in well-defined aspects of skill. One main argument in this framework is that deliberate practice is the most effective activity for improving performance and should therefore be maximized (36).

Deliberate play stands in contrast to deliberate practice and was introduced by Côté (37) to describe sport activities that take place in informal settings, often with flexible rules adapted by the participants to maximize enjoyment rather than immediate performance. Examples include street football (“løkkefotball” in Norwegian) and informal play in schoolyards, where children themselves decide when, how, and with whom to play. These activities are typically characterized by high enjoyment, varied and spontaneous game situations, and frequent opportunities to experiment with new solutions, even though they are not necessarily designed around explicit learning goals (41). In a comparison of the original papers on deliberate practice (36) and deliberate play (37), Güllich et al. (40) highlight various characteristics of each practice form. Effort in deliberate practice involves full attention and concentration, tasks near or beyond current capability, and high physical and maximal mental effort. This category is, according to Güllich et al. (40), not applicable in deliberate play. Their findings from a sample of 208 young athletes did not fully support some of the premises of the definitions of deliberate practice and deliberate play. For example, inherent enjoyment was high in both activities and did not differ. These findings support a more nuanced view in which “deliberate practice” and “deliberate play” are seen as ideal-typical endpoints on a continuum of practice activities, rather than strict categories.

In the present study, we therefore focus on the defining characteristics of specific practice activities, such as degree of coach involvement, level of structure, feedback, effort, and enjoyment, rather than assigning entire developmental pathways to a single label like deliberate practice or deliberate play. This approach allows us to exploit the richness of the training diary by identifying different ways of training within Jason's everyday football activity, including forms that blend elements of practice and play.

Several studies have documented that athletes who reach elite levels were involved in more playful activity and several supplementary sports during childhood, compared with non-elite athletes (12, 42). A meta-analysis of 51 studies (involving 6,096 athletes, including 772 of the world's top performers) found that senior world-class athletes who began their main sport early and specialized were the exception, not the rule (12). Güllich et al. (43) further reported that athletes with rapid early performance gains, likely due to high amounts of main-sport practice, were more common among the highest junior performers and senior national-class athletes than among senior world-class athletes. Together, these findings suggest that the long-term development of expertise is supported by a mixture of practice-like and play-like activities over time, rather than by maximizing deliberate practice alone.

### 2.2 Deliberate play and practice in football

In football, research has shown that a range of activity forms, from structured, coach-led practice to informal play and participation in other sports, can contribute to long-term development (23, 42, 43). Nevertheless, several studies have reported positive associations between hours spent in football-specific practice during youth and later performance levels (9, 20, 23–25, 44). Hornig et al. (24) found that players in Bundesliga (Germany) accumulated 4,264 (mean value) hours over 16 years before their debut. Bundesliga players differed from amateurs by playing more leisure football during childhood and more organized football during adolescence and adulthood. The authors summarized previous studies by stating that most elite footballers started playing during early childhood. At the age similar to the present study (13–14 year), they typically practiced moderate volumes of organized football of roughly 100–300 h per year, and 250–430 h per year for the age group 14–18 years. In addition, non-organized leisure football amounted to approximately 80%–260% in childhood and around 0%–70% in adolescence, and involvement in other sports corresponded to roughly 20%–80% (childhood) and 20%–60% (adolescence) of the organized football volume (24).

Previous studies are based on retrospective participation history questionnaires. Although this method is established as a reliable and valid method to survey athletes' participation history from childhood to adulthood (45), it presents some challenges. Remembering what was being practiced several years ago is difficult. Moreover, many studies have used a limited set of broad activity categories such as “team practice”, “individual practice”, and “play”, potentially masking important variation in aims, structure, and feedback within each category (21, 23). For example, Haugaasen et al. (20) found that Norwegian footballers had been engaged in about 3,500 h *more* of football-specific practice at the age of 18 years than players in the study by Helsen et al. (22) which included Belgian international players. Haugaasen et al. suggested that this discrepancy could reflect differences in data collection (e.g., the inclusion of school practice in their study), statistical procedures, or potential cultural differences between countries. It also illustrates how sensitive cumulative hour estimates are to how activities are defined and categorized.

In light of these limitations, the present study uses the deliberate practice–deliberate play distinction to interrogate the micro-structure and context of one player's daily football-related activities over an entire year, rather than to infer general pathways from retrospective recall. By combining time-based summaries with qualitative information from the training diary and interview, we aim to illustrate how different ways of training, from highly structured, coach-led sessions to self-organized football with friends, interact within a grassroots environment to support long-term development.

## 3 Materials and methods

### 3.1 Study design and case description

This article is part of a larger project on talent development in football based on a grassroots club where eight of sixteen players who started in a youth team in 2015 (U13), later signed professional contracts. By early 2026 two are playing in top tier leagues in England and Holland, three in the Norwegian Premier League, one on level 2, and one on level 3. One national team player (U15–U18) has quit football (because of health issues not related to sport). All sixteen players were interviewed, and three of the authors have insight into the activity these players performed (58).

This study adopts an intrinsic longitudinal single case study design (46). In intrinsic case study research, the case is examined because it is of particular interest in itself and enables a deeper understanding of a specific phenomenon, rather than because it represents a population or is intended to support generalizable claims. The purpose is therefore to optimize understanding of the case's particularity and situated meaning. The present study focuses on one player (“Jason”) and its forms of practice and physical activity in a grassroots football environment which followed during one full year at age 13. The analytical interest lies not in identifying a normative or generalizable pathway in youth football development, but in documenting and understanding how physical activity and practice were organized across formal and informal contexts in this specific instance. Based on the interviews with players and coaches from the larger project, we consider Jason's training load to be a little higher, but it did not differ significantly from that of the other players he spent a lot of time with at the age of 13.

In any respect, this is a single-case study providing in-depth knowledge on talent development processes in the specific context of a Norwegian youth team in grassroots football. The case was selected retrospectively because the player later became a professional footballer, which allows detailed examination of activity patterns within a developmental trajectory. Hence, the case provides detailed empirical insight into one developmental context, enabling readers to engage in what Stake terms “knowledge transfer” (46).

### 3.2 Data collection

Detailed data on the player's activities were recorded in a training diary over the course of the year 2016. At the beginning of the registrations, the player was 13 years and 2 months old, entering his second season with the team. The recording of the training diary was done by the first author, Jason's father. As a former elite handball player (142 games for the national team), he had kept a detailed training diary for many years about his own training and therefore has extensive experience in keeping training diaries. He started recording Jason's training activity each day and the respective activity that Jason conducted and observed many of the sessions. The player's involvement in recording the diary was limited to continuously explaining the content of the training sessions. The first author and Jason were in constant contact about the type and length of activity that Jason did. When the first author did not himself observe a session, he asked Jason for the details of the session, which helped to categories the type of activities into a systematic dairy logging.

The diary followed a detailed spreadsheet structure in a Microsoft Excel file, including one row each session. The columns were organized in the following way: A) day/date, B) category of activity (e.g., team training, unorganized football alone, unorganized football with friends), C) detailed information about the content of the session, D) relevant additional information (e.g., name of tournament, place, holidays), and E) duration of exercise. Columns F to O consist of total sums for each training category (from columns B–E). For more detailed insight into the player's activities outside the organized formats, the categories “unorganized football alone,” “unorganized football with friends,” and “individual activities outside football” were structured in columns P to Y. To address potential observer and classification bias, all authors had access to the original Excel dataset and the third and fourth author participated in reviewing the categorization of activities as part of the data processing. Inconsistencies and unclear classifications were discussed within the author team and resolved through joint agreement. This process functioned as an internal audit of the coding process.

### 3.3 Data analysis and validation

Descriptive statistics were applied, as presented in the Results section below. For validation purposes, Jason was interviewed by two co-authors (not the father), at the age of 22. The interview followed a timeline structure (47) where Jason acted as informant and was encouraged to tell his version about his journey. Thus, the structured timeline mapping method used in this interview was tailored to situations and contexts. The two underlying principles guiding the researcher are: (1) helping the participant in their recall process; and (2) fitting the visual (e.g., training diary) to the data the researcher plans to collect (48).

The interview was conducted via Teams, and the conversation was video and audio recorded and lasted for 46 min. Jason talked the co-authors through the timeline, and the co-authors posed probes, for example regarding how much he trained and how serious he was compared to the other boys on his team. During the conversation, the interviewers presented and continuously updated a visual timeline to mark Jason's football involvement, including different milestones, transitions, and other important information throughout his career so far. The timeline interview method was employed to facilitate Jason's recall of his football involvement, activity loads, club changes, and other important information throughout his development, as recommended by Adriansen (47). In the interview, Jason was encouraged to check, comment on, and if necessary, adjust the training diaries and records produced by his father to produce as accurate information as possible.

The interview was transcribed, and the first part of the analysis followed a deductive approach and included Jason's review of the training diaries and records, where he confirmed different estimates of hours of training activities and overall exercise loads throughout the period. There was a high degree of compliance between the numbers presented and Jason's recall. In the second part, the two authors conducting the interview used a combined deductive and inductive approach to analyze dynamic elements of activity, social dynamics, and personal development at different stages in Jason's football career. Typical deductively developed and analyzed questions asked were: “Are these amounts of training hours correct?” and/or “Is the estimation of the different organized football sessions correct?” When Jason was asked questions like: “How did this club change affect you as a football player?” and/or “What significance did a new coach have for your development as a footballer?” were these questions more inductively analyzed (48).

## 4 Findings

### 4.1 The amount and forms of activity

**Figure 1:**
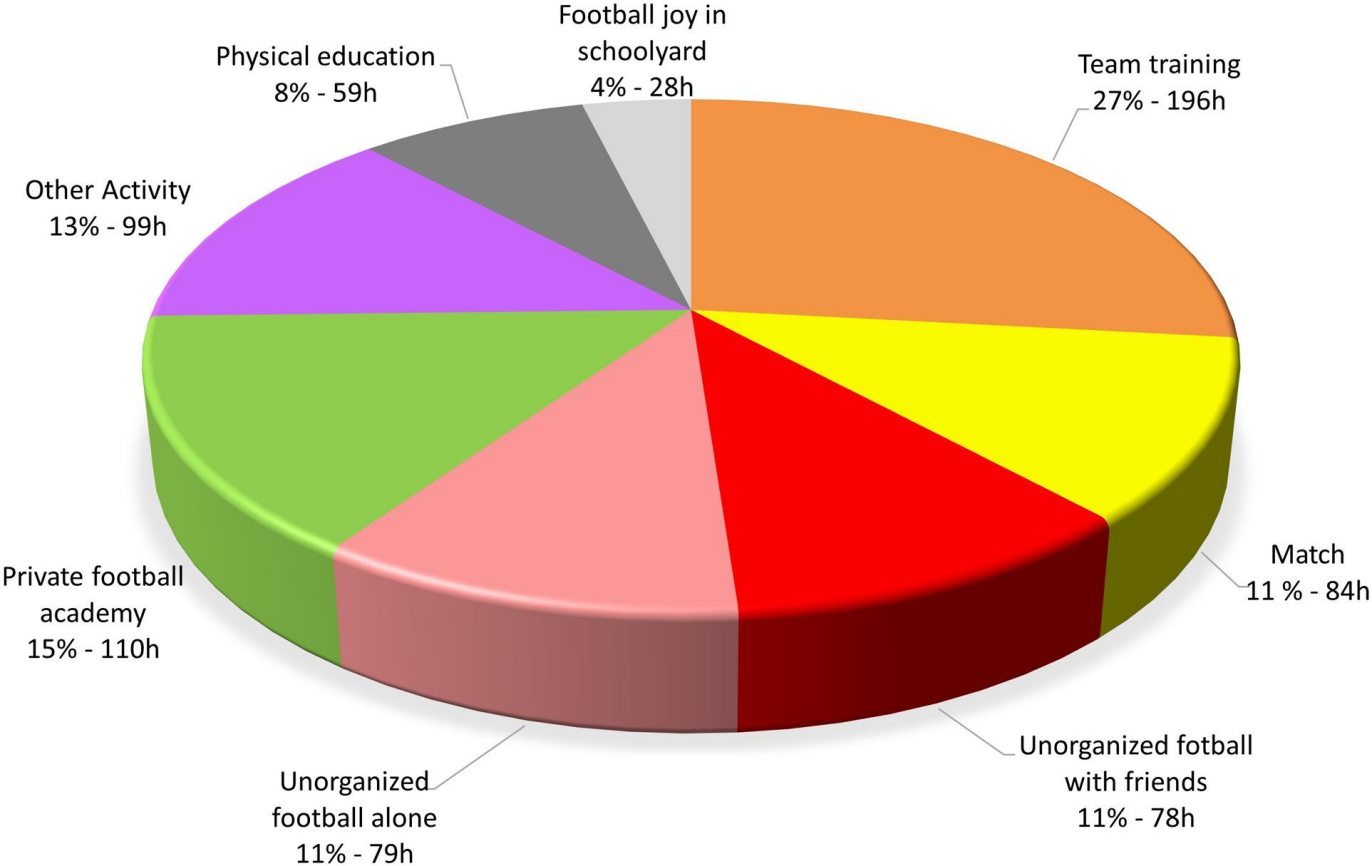
The different activities over one year (2016) for a 13-year-old football player who later became a professional at the highest level in Norway. Total: 733 h of activity.

Jason's total amount of activity in 2016 was 733 h. Figure 1 shows how this activity was divided into different categories. Football-specific activities dominated the total volume, accounting for 575 h (79%). The remaining 158 h (21%) consisted of physical education at school and activities categorized as “other” (e.g., strength and endurance training in various forms).

Most of the time was spent on organized football team training (196 h/129 sessions). This included his own club team (173 h/114 sessions) and weekly sessions organized by the regional football association (NFF Oslo) during the winter for selected players (the Landslagsskolen program; 23 h/15 sessions). A typical 90-minute of team training started with a 15–20 min warm-up that included injury-prevention component and simple passes, followed by 20–30 min of possession-oriented drills in small groups (e.g., 4v2 or 5v3) with constraints on touches and space to emphasize quick decision-making and ball circulation. The final part of the session consisted of games with different sizes of pitch and players on the teams (1v1, 3v3, or 5v5). At times the coaches joined in as players and provided feedback during short breaks rather than stopping playing continuously. For Jason, these sessions were experienced as both demanding and enjoyable, with clear performance-oriented goals but also a playful atmosphere.

Jason participated in 80 matches between January and November. The number may seem exceptional, but one explanation lies in the variety of match formats. Ordinary league matches in the Oslo region numbered 22, and unofficial matches 9. The format with the highest number of matches was tournaments (38), each consisting of 5–7 shorter games played over a weekend or a week.

Jason spent 110 h in a private academy, which accounted for 15 percent of his total activity. Almost all players on his grassroots club team participated in such private academies at age 11–12, but the number decreased when they turned 13–14. Jason continued because he found it both enjoyable and beneficial for his development. The concept at the private academy consisted of training in small groups that followed a set structure where four to five players were under guidance of a coach on a small pitch. The warm-up and initial exercises consisted of having as many touches on the ball as possible (the players were familiar with the exercise called “1000 touch”). All training had three components: scanning and awareness; decision-making with and without the ball, and development of purely technical skills. Each session lasted 75 min. In addition, there were some camps that took place over a weekend during the season.

Jason also played a lot of unorganized football. In 2016, this accounted for 22 percent of his total training. As shown in Figure 1, this activity is divided into three parts: unorganized football alone, unorganized football with friends, and football play in the schoolyard. Jason's self-organized sessions alone or with friends took place on one of the nearby pitches. The individual sessions (83 sessions, average 57 min) often focused on repeated passing against a wall, shooting and dribbling patterns he designed himself, sometimes inspired by exercises from team training or the private academy. These elements could also be included in the unorganized sessions with friends (57 sessions, 81 min). The boys organized the games themselves, rotating teams, positions, and formats such as 1v1, 2v2, 3v3, and they often adjusted pitch size and rules. Both the unorganized football alone and with friends were intrinsically motivating and self-chosen, but also contained several characteristics usually associated with deliberate practice, such as high repetition, focused attention, and work on identified weaknesses.

A proportion of children aged 12–13 still spend their school breaks engaged in various types of physical play. Jason and his classmates played football almost every day; this was estimated to approximately one hour per week, which is likely a conservative estimate. According to Jason, this activity was just for fun and not seen as training.

Physical education, which is a compulsory school subject, was estimated for each week. These hours include the elective subject “physical activity and health.” Other activities included different kinds of non-football training. At age 13, Jason began strength training and conducted some endurance and interval training. Outdoor activities, primarily with his family, were also included — such as cross-country skiing and cycling in the forest.

A typical week could look like the example from 19 to 25 September 2016, shown in Table 1. It consisted of 19 h, one and a half hours more than the average for the three-month period September–November (17.5 h per week). The table illustrates how different activity forms were interwoven in Jason's everyday life. On Monday and Tuesday, he combined unorganized football with friends, focusing on orientation, technical work, and small-sided games, with an evening team session that was described in the diary as “ordinary, mostly play” and followed by a short football-fitness strength circuit. Mid-week, he played a full league match at U14 level (100 min included warm-up), while Thursday included a private academy session in the morning (before school) with technical work and some football fitness as other activity in the evening. On Friday morning, he had an unorganized group session in football (2v2 games and crosses) and team training in the afternoon – a session he describes as “not intense”. This was opposite to Saturday with three sessions focusing on speed training. The same afternoon he played a competitive U14 match. The week completed with a resting day which included a walk in the woods with the family. The two rows at the bottom of Table 1 show accumulated time of physical activity and football joy in the schoolyard.

**Table 1:** A typical training week for jason, 19–25 September 2016 (m = morning; a = afternoon/evening). Total 19 h.

Day	Category	Details	Min.
Monday (a)	Unorg. football with friends	Perception, technical exercise	120
Tuesday (m)	Unorg. football with friends	Technique with “Peter”. High intensity	55
Tuesday (a)	Team training	A lot of games	80
Tuesday (a)	Other activity	Strength training/fitness	40
Wednesday	Match	Played full time	100
Thursday (m)	Private football academy	Ball control, 1v1	70
Thursday (a)	Other activity	Football fitness	40
Friday (m)	Unorg. football with friends	Technique, pass, game	90
Friday (a)	Team training	Not an intense session	90
Saturday (m)	Private football academy	Speed training, three sessions	180
Saturday (a)	Match	Played full time	100
Sunday	No training	Walk with family in the woods (10 km)	0
Week	Physical education	Three sessions (accumulated time)	120
Week	Football joy in schoolyard	Five days (accumulated time)	60
Total			1145

### 4.2 Variation during the year

**Figure 2:**
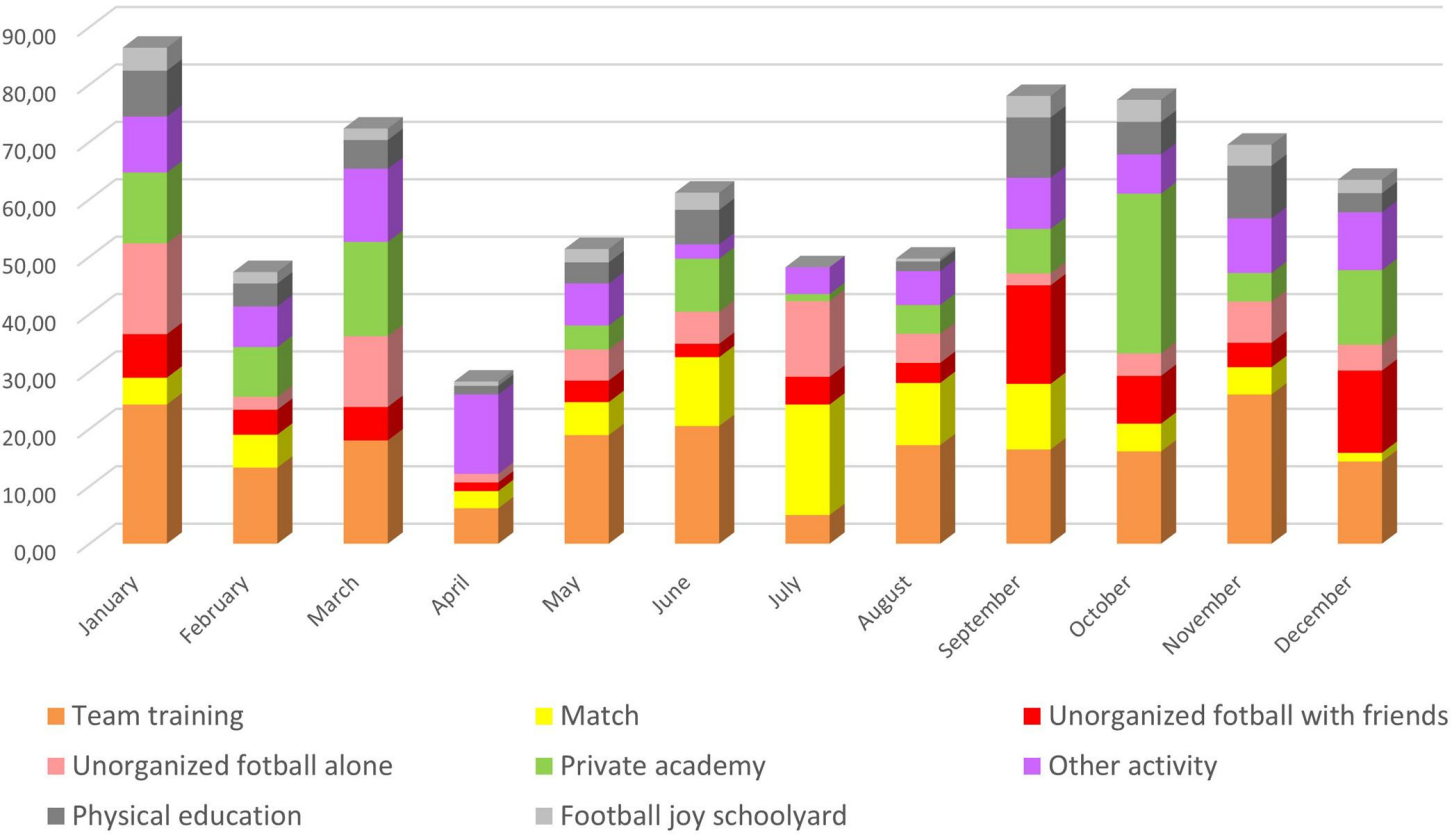
Activity divided by month and activity type for one year (2016) for a 13-year-old football player who later became a professional at the highest level in Norway (total 733 h of activity; see Supplementary Table 1 for monthly hours in each category).

In Norway, the football season starts in spring and ends in autumn. For youth teams, league matches typically take place from April to October, with a break during the school summer holiday (late June - mid-August). Historically, playing football outside the competitive season was challenging due to cold winters and snow in most parts of the country. However, the introduction of artificial turf pitches (of which some are heated or indoors) has turned football into a year-round sport. Figure 2 shows the distribution of activities for Jason across the year. The exact monthly hours for each activity category are reported in Supplementary Table 1.

In 2016, Jason's team began training in the first week of January and continued until December. The total of 733 training hours corresponds to an average of about 61 h per month, or roughly two hours per day. The highest training load occurred in January (86 h), while the lowest was recorded in April (42 h), which may seem surprising because spring is a period with a lot of football in Norway. The explanation lies in an injury – a common occurrence in youth football (49, 50). During a match in February, Jason suffered an ankle injury that reduced his training load for two weeks. He returned to team training, but an MRI scan in early April revealed fractures and cartilage damage in the ankle. He was prescribed two weeks of complete rest, followed by two more weeks on crutches (strength training was allowed). Despite the injury, he continued to train alternatively almost every day (categorized as “other activity” in Figure 2, which is high in March–April). He missed several team training sessions and the team's first league matches of the season but remained injury-free for the rest of the year.

As shown in Figure 2, the different activities varied throughout the season. Almost all activity types were represented each month. One striking observation is that total activity did not decline significantly during the summer holidays. Instead, match frequency increased considerably during this period. It is common for youth teams to participate in one or more tournaments during the school summer break. As shown in Figure 2, Jason played numerous matches during this period — an activity he particularly enjoyed. The boys on the team had established close friendships and strong group cohesion long before joining the U13 squad (58). Traveling together, staying in classrooms or hotels, and engaging in social activities made these tournaments memorable experiences in which football was just one of several important elements.

### 4.3 Accuracy, sobriety, and honesty

During the time-line driven interview Jason recalled and confirmed the presented data over the amount of training hours, the variation in different football training sessions conducted, and other milestones like club changes including new coaches and teammates. When studying the different numbers and estimations he typically exclaimed; “My father is not known for exaggerating, so this is probably correctly estimated.” Jason also emphasized the importance of club changes both at a vital time in his career and the importance and influence of new coaches for his development as a football player specifically and as a person in general. He spoke very frankly about why he chose to play for just that club at that time in his football career. He reported; “It was crucial for me to play at this level at this time.”

Further, the analysis generated a conscious link, as Jason explicitly articulated relationships between club changes, new environments, and his own adaptation. These environmental changes – involving new coaches, teammates, and other external structures – highlighted and clarified personal characteristics such as his eagerness to train, willingness to learn, and persistence and stubbornness.

## 5 Discussion

The aim of this paper is to examine the load and nature of physical activity both inside and outside organized football to better understand the football talent development process. As a point of departure, we address the difficulty of distinguishing between deliberate play and deliberate practice within talent development in team sports such as football. When Jason's annual training volume and distribution of activities are compared with retrospective participation histories of players who later reached professional or elite level (20, 22–24), they fall within, and in some periods slightly above, the ranges typically reported for this age group, particularly with respect to organized team practice and unorganized football. At the same time, our single-case design does not allow us to specify “how much is required” to become good at football, but rather to illustrate one empirically documented pathway within these broader ranges.

### 5.1 Organized football activities

As shown in Figure 1, training and matches with the team, together with participation in a private academy, accounted for over half of the total practice (52%). According to the definition of deliberate practice, such training is led by coach(es) as a highly structured activity, characterized by feedback and with an explicit goal of improving performance. In line with Ericsson's account of deliberate practice, these organized sessions were primarily designed and led by coaches, with explicit performance-improvement goals, high structure, and frequent opportunities for corrective feedback (36, 39). At the same time, not all team sessions were “pure” examples of deliberate practice but occupied different positions along a practice continuum where play-like elements were also present (40).

The practice consists of frequent repetitions and is physically and mentally demanding. Moving into detail, we find some nuances. Jason's training diary had notes for almost every team training session. The sessions followed a traditional structure with warm-up including injury prevention, possession-based exercises, specific tactical aspects, and game-like activities on small pitches organized as 1v1 or 3v3. The latter emphasized competition, creativity, intensity, and skill development. These small-sided games combined high physical demands with a strong element of enjoyment and can therefore be understood as hybrid forms that draw on both deliberate practice (targeted, effortful training) and deliberate play (spontaneous, enjoyable game situations (40, 41).

For the most part, the games during training did not involve detailed coaching. Instead, one or both coaches participated as players. Feedback was given during breaks and after the games. More detailed feedback on tactics and playing style was given during full-size training and matches.

Also included in the team training was futsal once a week during the winter. The coaches considered this form of indoor training important. One of them, a former national-team player, emphasized the importance of using futsal to impose a high cognitive load on players and to develop the ability to gather and use information (perception) (58). Technical execution without looking down at the ball frees up attentional capacity, allowing players to scan and interpret the environment more effectively (51).

Communication (verbal and non-verbal) and creativity were also key features of futsal activity (52, 53). The coach provided limited direct instruction, acting more as a role model and facilitator than as a constant instructor. The team had 12 futsal sessions organized by the club with coaches present. The same group of players also had eight self-organized futsal sessions at the same venue (logged as “unorganized football with friends” in the diary). Both types of sessions were characterized by joy and intrinsic engagement, elements often associated with deliberate play (41).

As shown in Figure 1, the team took part in a variety of match formats. Overall, the coaches prioritized development over results. In the league system for youth in Norway there is no promotion or relegation in U14, but the clubs register the team based on the level they consider reasonable. Jason's team played at the highest level in the Oslo region, but these matches were supplemented by tournaments on different levels, for example Norway Cup in Oslo (mass participation) and Audi Cup in Copenhagen (elite, by invitation). Another format was indoor or futsal tournaments with 3–5 players on the team (11 matches). These events were primarily characterized by enjoyment, often without coaches on the sidelines. In most league matches and elite tournaments, the coaches provided continuous, directive feedback from the sideline – constantly instructing the players (regarding movements and passes). Some players reported that this coaching style was demanding (58), which is in line with the high effort and concentration typical of deliberate practice (36). Overall, the matches represented a blend of serious and playful elements, reflecting the interplay of deliberate practice and deliberate play in football development.

Private academies have become increasingly common in Norway, particularly in the big cities (18). Jason attended an academy that targeted development of young football players as their main goal: “Small groups allow for close follow-up, frequent feedback, and a personal approach that promotes learning and mastery” (54). Here we find some similarities to the original deliberate practice literature by Ericsson et al. (36), which emphasizes high structure, tasks beyond current level, immediate corrective feedback, and high effort. These sessions are therefore close to the type of purposeful, individualized, high-effort training that skill-acquisition research identifies as particularly effective for developing specific aspects of performance (39, 55). At the same time, Jason's reports that he found the work enjoyable illustrates that highly structured practice need not be experienced as “unfun”, challenging simple dichotomies between practice and play (40).

While Ericsson (38) notes that training with *the whole team* cannot meet the criteria for individualized training (a key in deliberate practice), the private academy's small-group format approximates these criteria. Jason received more individualized feedback and closer follow-up at the academy than in the 15–20-player team environment. The personal training sessions lasted 75 min with Jason alone or more typically 4–5 players. The academy also held camps (1–3 days), with division of players into groups (5–8 players) based on skill levels. The program targeted enthusiastic, self-driven players, and the methodology could be considered advanced. All exercises contained three components: (a) scanning and vision, (b) decision-making, and (c) technical execution – a philosophy the academy continues to follow today (58). The first two elements were particularly important areas for improvements for Jason, and he found working on them enjoyable.

### 5.2 Unorganized football activities

In the training diary, this activity was divided into three parts: Unorganized football alone, unorganized football with friends, and football at school (in the schoolyard or in the school's sports hall). Jason considered football activities (alone or with friends) to be part of his training – something he engaged in to become a better football player. Hence, the activity was deliberate. In terms of our conceptual framework, many of these sessions fit well with the idea of deliberate play: they took place in informal settings, were self-organized by the players, involved flexible rules, and were primarily motivated by enjoyment and the intrinsic pleasure of playing (37, 41). Today, being a full-time professional footballer, with a workload that is too high to add informal sessions, he “…misses this kind of training, working individually or with friends on details to improve my performance” (Jason).

The activity categorized as unorganized football alone included 24 different activities across 83 sessions during the year, such as dribbling, shooting, ball control, first touch, and M-station (football rebounder). By systematizing the sessions, close ball control seems to have been the main objective of this training, followed by passing (against a wall), crossing, and shooting. These sessions alone were highly repetitive, goal-directed, and often focused on identified weaknesses, which places them close to deliberate practice even though they were self-planned, without any coaching and inherently enjoyable for Jason (36).

Unorganized football with friends had some of the same elements as football alone, for example ball control and passing. However, training with friends mainly included games in various formats, such as 1vs1, 2vs2, 3vs3, and games with more players on pitches of different sizes. Coaches were not involved, but the main coach observed many of the sessions from his office in the clubhouse and was struck by the high intensity and the strong desire to win. In his opinion, these guys didn't need a coach on the sidelines to work hard (58). These sessions are good examples of hybrid activities between deliberate play and deliberate practice: they were player-led, flexible, and highly enjoyable, but also involved sustained effort, frequent 1v1 and 2v2 duels, and a shared focus on “working hard” and improving skills (41, 43). Such hybrid forms fit well with recent arguments that practice and play should be understood as endpoints on a continuum of practice activities rather than mutually exclusive categories (43, 56).

This is illustrated in a note in the diary (23rd of October) under unorganized football with friends: “Juggling the ball and other things. Mostly play and fun due to lack of a pitch.” The session was registered as 0 min in the diary, which indicates that Jason did not see this as formal training. Second, lack of pitches was an exception: Jason and his friends always found a space to play football. According to the head of player development in NFF, Håkon Grøttland (57), a very important factor behind Norway's football development was the artificial turf and facility revolution around the turn of the millennium. In a Facebook post he explained that “Football suddenly became an all-year sport, and those who wanted to train more had access to good-quality pitches throughout the year.”

In Norway, other sports face challenges regarding facilities. For example, in their study on one of the best female handball players in the world, Haugen et al. (35) pointed out that her training volume during childhood and youth was lower than for football players, particularly when considering sport-specific training. The limited availability of handball courts, especially during the winter months, was the main reason.

In line with the literature on deliberate play, these unorganized football activities can be seen as essential for the development of intrinsic motivation, creativity, and adaptive decision-making (41). Unlike deliberate practice, which is coach-led, structured, and effortful (36), deliberate play typically takes place in informal settings, is enjoyable, and promotes game intelligence and tactical awareness. Taken together with the organized activities described above, the present case therefore illustrates how a mixture of practice-like and play-like forms, rather than a maximization of any single type, characterized Jason's developmental environment, consistent with recent synthesis work in expertise and talent development.

### 5.3 Limitations

Several limitations of the study should be acknowledged. First, the single-case design and the specific Norwegian context (strong club culture, extensive facility access, and a relatively regulated children's sport system) limit the generalizability of the findings to other settings and countries. Second, although the training diary was detailed and later validated through a quality check, the data still rely on one primary recorder (the player's father), which may introduce measurement and classification bias, particularly regarding the distinction between play and practice. Third, the study focuses on one year at age 13 and does not provide a full longitudinal account of the player's developmental trajectory from childhood to senior level.

## 6 Conclusions

This single-case study provides a rare, detailed account of one year of training and physical activity in a 13-year-old grassroots football player who later progressed to the highest professional level in Norway. The findings show that his developmental pathway was characterized by a substantial overall training volume, a high proportion of organized football-specific activity, and a considerable amount of unorganized football and other physical activities. The findings illustrate a nuanced interplay between deliberate practice and deliberate play, where coach-led team training, private academy sessions, and informal football with friends all contributed to his long-term development. Moreover, the study highlights how structural conditions in Norwegian grassroots sport – including accessible facilities, a strong club environment, and a culture that allows both sampling and later specialization – can support the emergence of elite players outside traditional academy pathways. These insights underline the importance of viewing talent development not as a linear accumulation of practice hours, but as a dynamic process in which play, practice, context, and motivation are deeply intertwined.

## Data Availability

The original contributions presented in the study are included in the article/Supplementary Material, further inquiries can be directed to the corresponding author.
