# Supplementary material for: The fine line between deliberate play and deliberate practice within talent development in grassroots football: evidence from a one-year longitudinal case study
Source: Front Sports Act Living. 2026 Apr 27;8:1799878. doi: 10.3389/fspor.2026.1799878 (PMC13158181; doi:10.3389/fspor.2026.1799878)
Supplement: Supplementary file 1 [file Table1.docx]

**Supplementary Table 1**. Monthly distribution of training hours across activity categories (team training, matches, unorganized football alone, unorganized football with friends, private academy, physical education, other activities, and football play in the schoolyard) for Jason in 2016. The bottom row shows the total annual hours in each activity category and in total.

| **Month** | **Team training** | **Match** | **Unorg. football with friends** | **Unorg. football alone** | **Private academy** | **Other activity** | **Physical education** | **Football joy in schoolyard** | **Total hours** |
| --- | --- | --- | --- | --- | --- | --- | --- | --- | --- |
| January | 24 | 5 | 8 | 16 | 12 | 10 | 8 | 4 | **86** |
| February | 13 | 6 | 4 | 2 | 9 | 7 | 4 | 2 | **47** |
| March | 18 | 0 | 6 | 12 | 16 | 13 | 5 | 2 | **72** |
| April | 6 | 3 | 2 | 2 | 0 | 14 | 2 | 1 | **28** |
| May | 19 | 6 | 4 | 5 | 4 | 7 | 4 | 2 | **51** |
| June | 20 | 12 | 2 | 6 | 9 | 2 | 6 | 3 | **61** |
| July | 5 | 19 | 5 | 13 | 1 | 5 | 0 | 0 | **48** |
| August | 17 | 11 | 4 | 5 | 5 | 6 | 2 | 0 | **50** |
| September | 16 | 11 | 17 | 2 | 8 | 9 | 10 | 4 | **78** |
| October | 16 | 5 | 8 | 4 | 28 | 7 | 6 | 4 | **77** |
| November | 26 | 5 | 4 | 7 | 5 | 10 | 9 | 4 | **70** |
| December | 14 | 2 | 14 | 4 | 13 | 10 | 3 | 2 | **63** |
| **Total category** | **196** | **84** | **78** | **79** | **111** | **99** | **59** | **28** | **733** |
